# Stroke-associated infection in patients with co-morbid diabetes mellitus is associated with in-hospital mortality

**DOI:** 10.3389/fnagi.2022.1024496

**Published:** 2022-12-01

**Authors:** Minping Wei, Qin Huang, Fang Yu, Xianjing Feng, Yunfang Luo, Tingting Zhao, Ruxin Tu, Di Liao, Yang Du, Qing Huang, Wenping Gu, Yunhai Liu, Yingyu Jiang, Hongqiu Gu, Zixiao Li, Jian Xia

**Affiliations:** ^1^Department of Neurology, Xiangya Hospital, Central South University, Changsha, China; ^2^Clinical Research Center for Cerebrovascular Disease of Hunan Province, Central South University, Changsha, China; ^3^National Clinical Research Center for Geriatric Disorders, Xiangya Hospital, Central South University, Changsha, China; ^4^Department of Neurology, Beijing Tiantan Hospital, Capital Medical University, Beijing, China; ^5^China National Clinical Research Center for Neurological Diseases, Beijing Tiantan Hospital, Capital Medical University, Beijing, China; ^6^National Center for Healthcare Quality Management in Neurological Diseases, Beijing Tiantan Hospital, Capital Medical University, Beijing, China

**Keywords:** infection, ischemic stroke, diabetes mellitus, in-hospital mortality, early poor functional outcome

## Abstract

**Background and objective:**

The association between infection and acute ischemic stroke (AIS) with diabetes mellitus (DM) remains unknown. Therefore, this study aimed to explore the effect of infection on AIS with DM.

**Materials and methods:**

The data of patients with AIS and DM were extracted from the Chinese Stroke Center Alliance (CSCA) database from August 2015 to July 2019. The association between infections [pneumonia or urinary tract infection (UTI)] and in-hospital mortality was analyzed. Logistic regression models were used to identify the risk factors for in-hospital mortality of patients with infection.

**Results:**

In total, 1,77,923 AIS patients with DM were included in the study. The infection rate during hospitalization was 10.5%, and the mortality rate of infected patients was 3.4%. Stroke-associated infection was an independent risk factor for an early poor functional outcome [odds ratio (OR) = 2.26, 95% confidence interval (CI): 1.97–2.34, *P* < 0.0001] and in-hospital mortality in AIS patients with DM. The in-hospital mortality after infection was associated with age (OR = 1.02, 95% CI: 1.01–1.03, *P* < 0.0001), male (OR = 1.39, 95% CI: 1.13–1.71, *P* = 0.0018), reperfusion therapy (OR = 2.00, 95% CI: 1.56–2.56, *P* < 0.0001), and fasting plasma glucose at admission (OR = 1.05, 95% CI: 1.03–1.08, *P* < 0.0001). In contrast, antiplatelet drug therapy (OR = 0.63, 95% CI: 0.50–0.78, *P* < 0.0001) and hospital stay (OR = 0.96, 95% CI: 0.94–0.97, *P* < 0.0001) were independent protecting factors against in-hospital mortality of patients with infection.

**Conclusion:**

Infection is an independent risk factor of in-hospital mortality for patients with AIS and DM, and those patients require strengthening nursing management to prevent infection.

## Introduction

Stroke is a serious and life-threatening disease and the second leading cause of death in the world (GBD 2019 Stroke Collaborators, 2021). Acute ischemic stroke (AIS) accounts for 62.4% of all stroke cases (GBD 2019 Stroke Collaborators, 2021). A study has shown that up to 9.6% of ischemic stroke (IS) patients developed an infection during hospitalization, with more than 95% of the infections being pneumonia and urinary tract infections (Zonneveld et al., 2017; Westendorp et al., 2018; Xu et al., 2020). Stroke-associated infection is not merely associated with increased mortality and longer hospital stays but is also an independent risk factor for early stroke recurrence (Kwan and Hand, 2007; Rocco et al., 2013; Xu et al., 2020).

According to the China Stroke Statistics 2019, about 23.5% of IS patients had diabetes mellitus (DM; Wang et al., 2020). DM is a common complication of stroke and is also an independent risk factor for IS (Tuttolomondo et al., 2008). Stroke patients with DM generally have a poor prognosis (Szlachetka et al., 2020; Yao et al., 2020). Evidence suggests that DM can increase the risk of pneumonia and urinary tract infection (UTI) in stroke patients (Liao et al., 2015). Admission hyperglycemia is associated with post-stroke infection, even in patients without DM (Zonneveld et al., 2017). A 7-year nationwide prospective research of 5,12,869 adults demonstrated an increased risk of death after infection in DM patients (Bragg et al., 2017). Impaired immune function during hyperglycemia may be involved in the increased susceptibility to infection and mortality in DM patients (Donnelly et al., 2017). In addition, in AIS patients, the activation of inflammatory factors at the infarct location, the immunosuppression induced by stroke, the immune exhaustion after the inflammatory response, and the sympathetic activation further promote immune disorders (Emsley and Hopkins, 2010; Shim and Wong, 2018). Considering the scarcity of epidemiological data on the effects of stroke-associated infection in patients with DM and IS and the increased poor prognosis observed with DM and infection in stroke patients, we suspected that stroke-associated infection during hospitalization might increase the risk of in-hospital mortality in patients with AIS and DM.

Therefore, the study aims to determine the effects of stroke-associated infection on early functional outcomes and in-hospital mortality in patients with AIS and DM and to identify independent risk factors for in-hospital mortality in these patients. The results will elucidate the relationship between stroke-associated infection, the risk of in-hospital mortality, and early poor functional outcomes in patients with AIS and DM, providing clinical evidence for strengthening nursing management.

## Materials and methods

### Patients

This study used data from a national prospective multicenter registration study, the Chinese Stroke Center Alliance (CSCA). All hospitals participating in the CSCA have received approval to collect data without obtaining individual patient informed consent or a waiver of authorization and exemption from their institutional review board. All procedures in this study conformed to the ethical standards of the institutional and/or national research council and the principles of the 1964 Declaration of Helsinki. A total of 1,77,923 patients diagnosed with AIS and DM from August 1, 2015 to July 31, 2019, were enrolled in the study. The inclusion criteria of our study were as follows: (1) age ≥18 years; (2) diagnosis of AIS confirmed by brain computed tomography or magnetic resonance imaging; (3) within 7 days of onset; (4) a previous history of DM. Patients with missing data, including in-hospital mortality, time from symptom onset to admission, or symptom onset beyond 7 days, were excluded. The diagnosis of DM was based on previous clinical medical records.

### Infection and baseline characteristics

In the following analyses, stroke-associated infections were defined as pneumonia and UTI in stroke patients during hospitalization (the most common stroke-associated infections), and the relevant data were obtained from discharge records (Davenport et al., 1996). The baseline characteristics included demographic characteristics, body mass index (BMI), blood pressure at admission, personal history, past medical history, medication history, National Institutes of Health Stroke Scale (NIHSS) score, modified Rankin Scale score (mRS), hospital stay, and biochemical data. The range of the NIHSS score is 0–42, with a higher score indicating a more severe neurological deficit (Yoshimura et al., 2022). The range of mRS score is 0–6, with 0 indicating no disability, 6 death and a higher score indicating greater disability (Yoshimura et al., 2022). The NIHSS and mRS scores were rated by a trained professional neurologist and recorded in the inpatient medical records.

### Outcome

The primary outcome was all-cause death during hospitalization, i.e., in-hospital mortality. The secondary outcome was the early poor functional outcome, defined as discharge mRS score 3–6 (Boehme et al., 2016).

### Statistical analyses

Measurement data were presented as means ± standard deviation (SD) or medians with interquartile ranges (IQR). Enumeration data were presented as numbers and percentages. The Mann–Whitney u-test was used to compare continuous data that did not comply with a normal distribution, while the student’s *t*-test was used to compare continuous data complying with a normal distribution. The Chi-Squared test was used to compare the proportions of categorical variables. Univariate and multivariable logistic regression analyses were used to estimate the relationship between stroke-associated infection (including total infection, pneumonia, and UTI) and early poor functional outcomes and in-hospital mortality. Univariate and multivariable logistic regression analyses were also used to identify risk factors for in-hospital mortality after infection in patients with AIS and DM. The odds ratio (OR) was presented with the corresponding 95% confidence interval (CI). All statistical analyses were performed using SAS 9.4 (SAS Institute, Cary, NC, USA). A *P*-value <0.05 (bilateral) was considered statistically significant.

## Results

### Population characteristics

**FIGURE 1:**
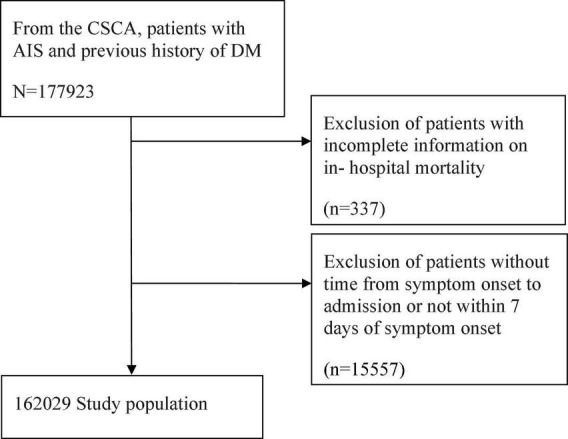
Flowchart of subject selection. CSCA, Chinese Stroke Center Alliance; AIS, acute ischemic stroke; DM, diabetes mellitus.

A total of 1,77,923 AIS patients with DM were registered in the CSCA database. However, 337 patients were excluded due to incomplete information on in-hospital mortality, and 15,557 patients were excluded as the time from symptom onset to admission was unavailable or exceeded 7 days from symptom onset. Finally, 1,62,029 patients were included in this study (Figure 1). The median age of all patients was 66 (IQR, 59–74) years, and 92,375 (57%) patients were male. Infection was found in 17,055 (10.5%) of the 1,62,029 patients (Table 1).

**TABLE 1:** Demographic and clinical characteristics of patients.

Variables*	Total (*n* = 162029, 100%)	Non-infection (*n* = 144974, 89.5%)	Infection (*n* = 17055, 10.5%)	*P*-value
Age, years	66 (59–74)	66 (58–73)	72 (64–79)	< 0.0001
Male	92375 (57.0)	83477 (57.6)	8898 (52.2)	< 0.0001
BMI, kg/m^2^	24.2 (22.3–26.0)	24.2 (22.4–26.0)	23.9 (21.8–26.0)	< 0.0001
SBP, mmHg	151.3 ± 22.4	151.1 ± 22.2	152.9 ± 24.3	< 0.0001
DBP, mmHg	86.1 ± 13.1	86.1 ± 12.9	85.7 ± 14.2	< 0.0001
Previous TIA	2476 (1.5)	2179 (1.5)	297 (1.7)	0.0164
Previous IS	61524 (38.0)	53766 (37.1)	7758 (45.5)	< 0.0001
Previous ICH	3811 (2.4)	3245 (2.2)	566 (3.3)	< 0.0001
Previous SAH	443 (0.3)	376 (0.3)	67 (0.4)	0.0016
Previous MI	4266 (2.6)	3575 (2.5)	691 (4.1)	< 0.0001
Hypertension	123791 (76.4)	110227 (76.0)	13564 (79.5)	< 0.0001
Dyslipidemia	23581 (14.6)	20546 (14.2)	3035 (17.8)	< 0.0001
Atrial fibrillation/flutter	7614 (4.7)	5584 (3.9)	2030 (11.9)	< 0.0001
Heart failure	1959 (1.2)	1322 (0.9)	637 (3.7)	< 0.0001
COPD	1389 (0.9)	881 (0.6)	508 (3.0)	< 0.0001
Current smokers	31724 (19.6)	29236 (20.2)	2488 (14.6)	< 0.0001
Alcohol drinkers	33715 (20.8)	30786 (21.2)	2929 (17.2)	< 0.0001
Reperfusion therapy	11205 (6.9)	9496 (6.6)	1709 (10.0)	< 0.0001
Antiplatelet	138125 (85.2)	124419 (85.8)	13706 (80.4)	< 0.0001
Anticoagulation	7708 (4.8)	6217 (4.3)	1491 (8.7)	< 0.0001
Lipid-lowering drug	11296 (7.0)	10167 (7.0)	1129 (6.6)	0.0565
NIHSS score at admission	3 (2–6)	3 (2–6)	7 (3–14)	< 0.0001
mRS score ≥3	29647 (18.3)	24131 (16.6)	5516 (32.3)	< 0.0001
Hospital stay, day	12 (8–14)	11 (8–14)	13 (9–19)	< 0.0001
CRP, mg/L	2.8 (1.0–6.5)	2.5 (0.9–6.0)	6.2 (2.0–21.9)	< 0.0001
LDL, mmol/L	2.7 (2.1–3.4)	2.7 (2.1–3.4)	2.6 (2.0–3.4)	< 0.0001
FPG at admission, mmol/L	8.2 (6.4–10.9)	8.1 (6.4–10.8)	8.7 (6.7–11.9)	< 0.0001
HbAlc,%	7.6 (6.5–9.2)	7.6 (6.5–9.2)	7.6 (6.5–9.3)	0.0602
HCY, mmol/L	13.0 (10.0–17.6)	13.0 (9.9–17.5)	13.4 (10.0–18.4)	< 0.0001

BMI, body mass index; SBP, systolic blood pressure; DBP, diastolic blood pressure; TIA, transient ischemic attacks; IS, ischemic stroke; ICH, intracerebral hemorrhage; SAH, subarachnoid hemorrhage; MI, myocardial infarction; COPD, chronic obstructive pulmonary disease; NIHSS, National Institutes of Health Stroke Scale; mRS, modified Rankin Scale score; CRP, C-reactive protein; LDL, low-density lipoprotein; FPG, fasting plasma glucose; HbAlc, hemoglobin A1c; HCY, homocysteine. *Continuous variables were expressed as means ± standard deviation or median (interquartile range), and classifying variables were expressed as numbers (percentages).

### Stroke-associated infection and early poor functional outcome

Table 1 displays the baseline characteristics of the patients included in the study. The results show that compared with patients without infection, the infected patients were older [median, 72 (IQR, 64–79) vs. 66 (IQR, 58–73), *P* < 0.0001], had a lower proportion of male patients (52.2 vs. 57.6%, *P* < 0.0001), had higher admission systolic blood pressure (SBP) (*P* < 0.0001), had lower BMI (*P* < 0.0001), had more underlying diseases such as hypertension (*P* < 0.0001), dyslipidemia (*P* < 0.0001), atrial fibrillation/flutter (*P* < 0.0001), heart failure (*P* < 0.0001), and chronic obstructive pulmonary disease (COPD) (*P* < 0.0001). Furthermore, patients with infection demonstrated higher rates of previous cardiovascular and cerebrovascular events, including transient ischemic attacks (TIA) (*P* = 0.0164), IS (*P* < 0.0001), intracerebral hemorrhage (ICH) (*P* < 0.0001), subarachnoid hemorrhage (SAH) (*P* = 0.00016), and myocardial infarction (MI) (*P* < 0.0001), and had a higher NIHSS score at admission [median, 7 (IQR, 3–14) vs. 3 (IQR, 2–6), *P* < 0.0001]. Regarding blood chemical indexes, infected patients had higher levels of C-reactive protein (CRP) [median, 6.2 (IQR, 2.0–21.9) vs. 2.5 (IQR, 2.0–21.9), *P* < 0.0001], fasting plasma glucose (FPG) at admission [median, 8.7 (IQR, 6.7–11.9) vs. 8.1 (IQR, 6.4–10.8), *P* < 0.0001], and homocysteine (HCY) (*P* < 0.0001). In addition, patients with infection were more likely to be treated with reperfusion therapy (10.0 vs. 6.6%, *P* < 0.0001) and anticoagulation (8.7 vs. 4.3%, *P* < 0.0001) but less likely to be treated with antiplatelets (80.4 vs. 85.8%, *P* < 0.0001).

Remarkably, infected patients not only had longer hospital stays [median, 13 (IQR, 9–19) vs. 11 (IQR, 8–14), *P* < 0.0001) but also had a higher proportion of patients with early poor functional outcome (mRS score ≥3 of hospital discharge) (32.3 vs. 16.6%, *P* < 0.0001, Table 1). The multivariable logistic regression analysis revealed that stroke-associated infection is an independent risk of early poor functional outcomes in AIS patients with DM (OR = 2.26, 95% CI: 1.97–2.34, *P* < 0.0001, Table 2). In subgroup analysis, pneumonia (OR = 2.15, 95% CI: 2.06–2.48, *P* < 0.0001) and UTI (OR = 1.82, 95% CI: 1.50–2.22, *P* < 0.0001) were independent risk factors.

**TABLE 2:** Association between stroke-associated infection and early poor functional outcome.

Covariate	Unadjusted OR (95% CI)	*P*-value	Adjusted OR (95% CI)	*P*-value
Infection	4.40 (4.19–4.62)	<0.0001	2.15(1.97–2.34)	<0.0001
Pneumonia	4.82(4.57–5.08)	<0.0001	2.26(2.06–2.48)	<0.0001
UTI	2.76(2.46–3.07)	<0.0001	1.82(1.50–2.22)	<0.0001

Adjusted for age, sex, BMI, NIHSS score, SBP, DBP, hypertension, atrial fibrillation/flutter, heart failure, previous TIA, previous IS, previous MI, previous SAH, COPD, antiplatelet, anticoagulation, lipid-lowering drug, reperfusion therapy, smoking, alcohol, LDL, FBG, HbA1c, HCY.

### Stroke-associated infection and in-hospital mortality

Table 3 shows the baseline characteristics of the infected patients subgrouped by mortality. Among 17,055 infected patients with AIS and DM, 574 (3.4%) cases resulted in in-hospital death. Compared with patients who survived, the dead patients were older [median, 76 (IQR, 66–82) vs. 72 (IQR, 64–79), *P* < 0.0001], had a higher proportion of male patients (57.0 vs. 52.0%, *P* = 0.0193), had higher admission SBP (*P* < 0.0001), had more underlying diseases, including hypertension (*P* = 0.0179), atrial fibrillation/flutter (*P* < 0.0001), and heart failure (*P* < 0.0001), and had a higher frequency of previous cardiovascular and cerebrovascular events, such as IS (*P* = 0.0137), SAH (*P* = 0.0110), and MI (*P* < 0.0001). Moreover, the mortality subgroup demonstrated more severe neurological dysfunctions NIHSS score at admission, [median, 16 (IQR, 10–23) vs. 7 (IQR, 3–13), *P* < 0.0001]. Regarding blood chemical indexes, the dead patients had higher CRP levels [median, 12.0 (IQR, 3.0–54.9) vs. 6.1 (IQR, 2.0–21.2), *P* = 0.0033], FPG at admission [median, 10.5 (IQR, 7.8–14.1) vs. 8.6 (IQR, 6.6–11.8), *P* < 0.0001], and HCY [median, 14.8 (IQR, 10.0–20.7) vs. 13.4 (IQR, 10.0–18.3), *P* = 0.0052]. Regarding treatment, patients with infection were more likely to be treated with reperfusion therapy (*P* < 0.0001) but less likely to be treated with antiplatelets (*P* < 0.0001) and lipid-lowering drugs (*P* = 0.0264).

**TABLE 3:** Demographic and clinical characteristics of infected patients.

Variables*	Total (*N* = 17055, 100%)	Non-death (*N* = 16481, 96.6%)	Death (*N* = 574, 3.4%)	*P*-value
Age, years	72 (64–79)	72 (64–79)	76 (66–82)	< 0.0001
Male	8898 (52.2)	8571 (52.0)	327 (57.0)	0.0193
BMI, kg/m^2^	23.9 (21.8–26.0)	23.9 (21.8–26.0)	23.7 (21.5–26.1)	0.2931
SBP, mmHg	152.9 ± 24.3	152.9 ± 24.2	154.5 ± 27.0	0.1482
DBP, mmHg	85.7 ± 14.2	85.6 ± 14.1	86.2 ± 15.6	0.3936
Previous TIA	297 (1.7)	287 (1.7)	10 (1.7)	0.9989
Previous IS	7758 (45.5)	7468 (45.3)	290 (50.5)	0.0137
Previous ICH	566 (3.3)	546 (3.3)	20 (3.5)	0.8217
Previous SAH	67 (0.4)	61 (0.4)	6 (1.0)	0.0110
Previous MI	691 (4.1)	626 (3.8)	65 (11.3)	< 0.0001
Hypertension	13564 (79.5)	13085 (79.4)	479 (83.4)	0.0179
Dyslipidemia	3035 (17.8)	2932 (17.8)	103 (17.9)	0.9244
Atrial fibrillation/flutter	2030 (11.9)	1867 (11.3)	163 (28.4)	< 0.0001
Heart failure	637 (3.7)	582 (3.5)	55 (9.6)	< 0.0001
COPD	508 (3.0)	485 (2.9)	23 (4.0)	0.1404
Current smokers	2488 (14.6)	2415 (14.7)	73 (12.7)	0.1434
Alcohol drinkers	2929 (17.2)	2823 (17.1)	106 (18.5)	0.4034
Reperfusion therapy	1709 (10.0)	1594 (9.7)	115 (20.0)	< 0.0001
Antiplatelet	13706 (80.4)	13347 (81.0)	359 (62.5)	< 0.0001
Anticoagulation	1491 (8.7)	1432 (8.7)	59 (10.3)	0.1849
Lipid-lowering drug	1129 (6.6)	1104 (6.7)	25 (4.4)	0.0264
NIHSS score at admission	7 (3–14)	7 (3–13)	16 (10–23)	< 0.0001
Hospital stay, day	13 (9–19)	14 (9–19)	7 (3–14)	< 0.0001
CRP, mg/L	6.2 (2.0–21.9)	6.1 (2.0–21.2)	12.0 (3.0–54.9)	0.0033
LDL, mmol/L	2.6 (2.0–3.4)	2.6 (2.0–3.4)	2.6 (1.8–3.4)	0.8156
FPG at admission, mmol/L	8.7 (6.7–11.9)	8.6 (6.6–11.8)	10.5 (7.8–14.1)	< 0.0001
HbAlc,%	7.6 (6.5–9.3)	7.6 (6.5–9.3)	7.6 (6.5–9.4)	0.8192
HCY, mmol/L	13.4 (10.0–18.4)	13.4 (10.0–18.3)	14.8 (10.0–20.7)	0.0052

BMI, body mass index; SBP, systolic blood pressure; DBP, diastolic blood pressure; TIA, transient ischemic attacks; IS, ischemic stroke; ICH, intracerebral hemorrhage; SAH, subarachnoid hemorrhage; MI, myocardial infarction; COPD, chronic obstructive pulmonary disease; NIHSS, National Institutes of Health Stroke Scale; mRS, modified Rankin Scale score; CRP, C-reactive protein; LDL, low-density lipoprotein; FPG, fasting plasma glucose; HbAlc, hemoglobin A1c; HCY, homocysteine. *Continuous variables were expressed as means ± standard deviation or median (interquartile range), and classifying variables were expressed as numbers (percentages).

The significant differences in previous SAH (*P* = 0.0108), hypertension (*P* = 0.0227), reperfusion therapy (*P* < 0.0001), lipid-lowering drug therapy (*P* = 0.0359), antiplatelet therapy (*P* < 0.0001), hospital stay (*P* < 0.0001), and CRP (*P* = 0.0053) between the mortality and survivor groups were mainly observed in patients with pneumonia. In contrast, the significant differences in gender (*P* = 0.0046), previous TIA (*P* = 0.0008), and therapy with anticoagulation (*P* = 0.0053) between the mortality and survivor groups were mainly found in patients with UTI (Table 4).

**TABLE 4:** Clinical features after infection in patients with pneumonia or urinary tract infection.

Variables*	Pneumonia				Urinary tract infection			
characteristics	Total (*N* = 15037)	Non-death (*N* = 14472, 96.2%)	Death (*N* = 565, 3.8%)	*P*-value	Total (*N* = 2,866)	Non-death (*N* = 2801, 97.7%)	Death (*N* = 65, 2.3%)	*P*-value
Age, years	72 (64–79)	72 (64–79)	76 (66–82)	< 0.0001	71(64–78)	71 (64–78)	78 (71–82)	0.0003
Male	8330 (55.4)	8006 (55.3)	324 (57.3)	0.3422	904 (31.5)	873 (31.2)	31 (47.7)	0.0046
BMI, kg/m^2^	23.8 (21.8–25.9)	23.8 (21.8–25.9)	23.7 (21.5–26.1)	0.2750	24.0 (21.9–26.3)	24.0 (21.9–26.3)	23.9 (20.8–26.6)	0.1255
SBP, mmHg	153.0 ± 24.4	153.0 ± 24.3	154.7 ± 26.8	0.1310	152.1 ± 24.4	152.2 ± 24.3	149.0 ± 29.8	0.3897
DBP, mmHg	85.8 ± 14.3	85.7 ± 14.2	86.3 ± 15.5	0.4319	84.9 ± 13.7	84.9 ± 13.6	84.6 ± 19.3	0.9054
Previous TIA	255 (1.7)	246 (1.7)	9 (1.6)	0.8469	71 (2.5)	67 (2.4)	4 (6.2)	0.0008
Previous IS	6803 (45.2)	6521 (45.1)	282 (49.9)	0.0230	1436 (50.1)	1390 (49.6)	46 (70.8)	0.1018
Previous ICH	498 (3.3)	479 (3.3)	19 (3.4)	0.9449	110 (3.8)	105 (3.7)	5 (7.7)	0.6127
Previous SAH	60 (0.4)	54 (0.4)	6 (1.1)	0.0108	11 (0.4)	11 (0.4)	0 (0)	0.6127
Previous MI	632 (4.2)	568 (3.9)	64 (11.3)	< 0.0001	118 (4.1)	107 (3.8)	11 (16.9)	< 0.0001
Hypertension	11965 (79.6)	11494 (79.4)	471 (83.4)	0.0227	2293 (80.0)	2238 (79.9)	55 (84.6)	0.3474
Dyslipidemia	2678 (17.8)	2576 (17.8)	102 (18.1)	0.8773	551 (19.2)	534 (19.1)	17 (26.2)	0.1516
Atrial fibrillation/flutter	1888 (12.6)	1729 (11.9)	159 (28.1)	< 0.0001	296 (10.3)	275 (9.8)	21 (32.3)	< 0.0001
Heart failure	594 (4.0)	539 (3.7)	55 (9.7)	< 0.0001	106 (3.7)	95 (3.4)	11 (16.9)	< 0.0001
COPD	487 (3.2)	464 (3.2)	23 (4.1)	0.2547	44 (1.5)	42 (1.5)	2 (3.1)	0.3065
Current smokers	2299 (15.3)	2226 (15.4)	73 (12.9)	0.2044	261 (9.1)	256 (9.1)	5 (7.7)	0.8586
Alcohol drinkers	2713 (18.0)	2610 (18.0)	103 (18.2)	0.9057	309 (10.8)	298 (10.6)	11 (16.9)	0.1063
Reperfusion therapy	1570 (10.4)	1455 (10.1)	115 (20.4)	< 0.0001	232 (8.1)	226 (8.1)	6 (9.2)	0.7341
Antiplatelet	11983 (79.7)	11628 (80.3)	355 (62.8)	< 0.0001	2367 (82.6)	2318 (82.8)	49 (75.4)	0.1213
Anticoagulation	1370 (9.1)	1313 (9.1)	57 (10.1)	0.4104	243 (8.5)	231 (8.2)	12 (18.5)	0.0035
Lipid-lowering drug	988 (6.6)	963 (6.7)	25 (4.4)	0.0359	196 (6.8)	191 (6.8)	5 (7.7)	0.7827
NIHSS score at admission	8 (4–14)	8 (3–14)	16 (10–23)	< 0.0001	6 (3–11)	6 (2–11)	20 (12–27)	< 0.0001
Hospital stay, day	13 (9–19)	14 (9–19)	7 (3–14)	< 0.0001	14 (10–19)	14 (10–19)	13 (4–30)	0.5621
CRP, mg/L	6.8 (2.1–23.0)	6.7 (2.1–22.4)	12.0 (3.0–54.9)	0.0053	5.0 (1.7–19.6)	5.0 (1.7–18.0)	30.0 (1.0–57.4)	0.0855
LDL, mmol/L	2.6 (2.0–3.4)	2.6 (2.0–3.4)	2.6 (1.8–3.4)	0.9189	2.7 (2.0–3.5)	2.7 (2.0–3.5)	2.4 (1.5–3.6)	0.2042
FPG at admission, mmol/L	8.7 (6.7–11.9)	8.6 (6.6–11.8)	10.5 (7.8–14.1)	< 0.0001	8.7 (6.6–11.9)	8.7 (6.6–11.8)	11.1 (7.9–14.0)	0.0041
HbAlc, %	7.6 (6.5–9.3)	7.6 (6.5–9.2)	7.6 (6.5–9.4)	0.5157	7.8 (6.5–9.6)	7.8 (6.5–9.6)	7.3 (6.5–9.1)	0.2077
HCY, mmol/L	13.5 (10.0–18.5)	13.5 (10.0–18.5)	15.0 (10.0–20.7)	0.0078	13.1 (9.6–18.0)	13.0 (9.6–18.0)	16.1 (12.0–25.0)	0.0004

BMI, body mass index; SBP, systolic blood pressure; DBP, diastolic blood pressure; TIA, transient ischemic attacks; IS, ischemic stroke; ICH, intracerebral hemorrhage; SAH, subarachnoid hemorrhage; MI, myocardial infarction; COPD, chronic obstructive pulmonary disease; NIHSS, National Institutes of Health Stroke Scale; CRP, C-reactive protein; LDL, low-density lipoprotein; FPG, fasting plasma glucose; HbAlc, hemoglobin A1c; HCY, homocysteine. *Continuous variables were expressed as means ± standard deviation or median (interquartile range), and classifying variables were expressed as numbers (percentages).

Next, the relationship between stroke-associated infection and in-hospital mortality was analyzed. Multiple logistic regression analysis revealed an independent association between stroke-associated infection and in-hospital mortality in AIS patients with DM (OR = 5.22, 95% CI: 3.74–7.28, *P* < 0.0001). In the subgroup analysis, pneumonia (OR = 5.81, 95% CI: 4.16–8.11, *P* < 0.0001) and UTI (OR = 2.52, 95% CI: 1.36–4.67, *P* = 0.0032) were identified as independent risk factors (Table 5).

**TABLE 5:** Association between post-stroke infection and in-hospital mortality.

Covariate	Unadjusted OR (95% CI)	*P*-value	Adjusted OR (95% CI)	*P*-value
Infection	14.56(12.72–16.65)	<0.0001	5.22(3.74–7.28)	< 0.0001
Pneumonia	16.12(14.10–18.43)	<0.0001	5.81(4.16–8.11)	< 0.0001
UTI	4.30(3.33–5.55)	<0.0001	2.52(1.36–4.67)	0.0032

Adjusted for age, sex, BMI, NIHSS score, SBP, DBP, hypertension, atrial fibrillation/flutter, heart failure, previous TIA, previous IS, previous MI, previous SAH, COPD, antiplatelet, anticoagulation, lipid-lowering drug, reperfusion therapy, smoking, alcohol, LDL, FBG, HbA1c, HCY. UTI, urinary tract infection.

### Analysis of risk factors for in-hospital mortality

**FIGURE 2:**
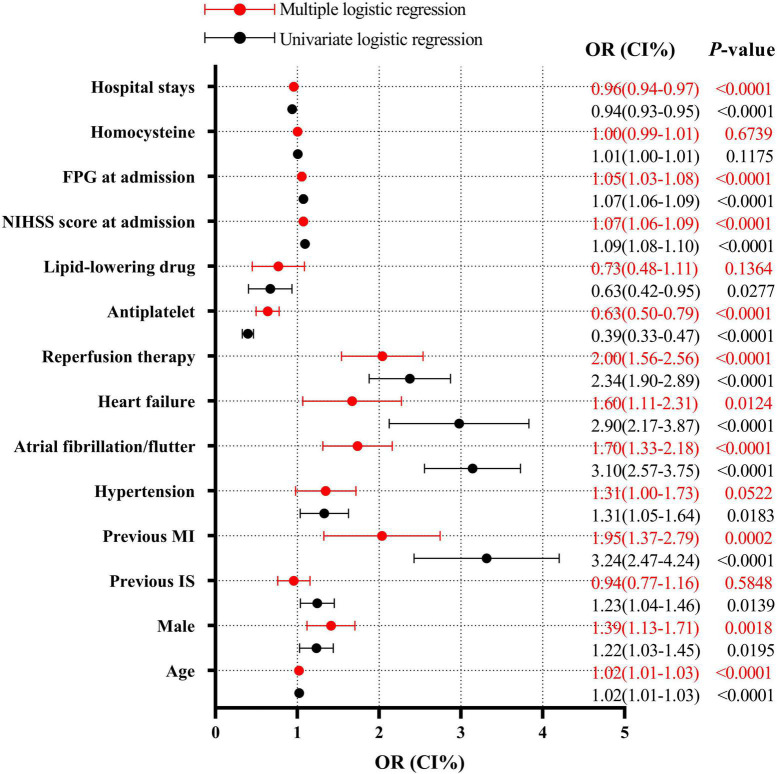
Logistic regression analysis for mortality after infection. FPG, fasting plasma glucose; NIHSS, National Institutes of Health Stroke Scale; MI, myocardial infarction; IS, ischemic stroke.

Further multiple regression analysis indicates that age (OR = 1.02, 95% CI: 1.01–1.03, *P* < 0.0001), male genderC (OR = 1.39, 95% CI: 1.13–1.71, *P* = 0.0018), previous MI (OR = 1.95, 95% CI: 1.37–2.78, *P* = 0.0002), atrial fibrillation/flutter (OR = 1.70, 95% CI: 1.33–2.18, *P* < 0.0001), heart failure (OR = 1.56, 95% CI: 1.11–2.31, *P* = 0.0124), reperfusion therapy (OR = 2.00, 95% CI: 1.56–2.56, *P* < 0.0001), NIHSS score at admission (OR = 1.07, 95% CI: 1.06–1.09, *P* < 0.0001), and FPG at admission (OR = 1.05, 95% CI: 1.03–1.08, *P* < 0.0001) were all the independent risk factors of in-hospital mortality in AIS patients with DM and infection. However, antiplatelet drug therapy (OR = 0.63, 95% CI: 0.50–0.78, *P* < 0.0001) and hospital stay (OR = 0.96, 95% CI: 0.94–0.97, *P* < 0.0001) were independent protecting factors against in-hospital mortality in AIS patients with DM and infection (Figure 2).

## Discussion

Our study provided solid and convincing evidence for the relationship between stroke-associated infection and patients with AIS and DM. Stroke-associated infection was found in 10.5% of patients with AIS and DM, and the rate of in-hospital mortality in patients with stroke-associated infection was 3.4%. Stroke-associated infection was an independent risk factor of early poor functional outcome and in-hospital mortality in patients with AIS and DM.

Xu et al. (2020) analyzed data from the CSCA database and found that the rate of stroke-associated infection was 9.6%, and the rate of in-hospital mortality in stroke patients with infection was 2.6%, which were all lower than the results of our study. Since our study population included AIS patients with DM, the disparity in results may indicate that DM patients are more likely to suffer from infection after AIS and more likely to die during hospitalization after infection. However, in 2004, data from large German stroke registers showed that post-stroke infections (namely pneumonia) contributed to in-hospital death in up to 31.2% of stroke patients (Heuschmann et al., 2004). In 2009, data from a small sample study in the Netherlands showed that 21% of patients with stroke-associated infection had died in the hospital, and 82% had a poor outcome at discharge (Vermeij et al., 2009). Although the AIS patients in our study were all complicated by DM, the in-hospital mortality of patients with stroke-associated infection was much lower than that of previous studies, which may reflect the gradual improvement of medical care, leading to a decline in mortality in stroke patients with infection.

Our results showed a greater risk of stroke-associated infection in females compared to male patients with AIS and DM, which was consistent with previous studies in AIS patients without DM (Suda et al., 2018). However, further analysis revealed that the patients who died after infection were more likely to be male and that the male gender is an independent risk factor of in-hospital mortality in patients with AIS and DM. Hormones may be responsible for the increased mortality risk. Research showed that estrogen could inhibit inflammation by reducing proinflammatory cytokines such as interleukin-1β (IL-1β) and reducing the cytotoxicity of natural killer cells. In contrast, androgens like testosterone could reduce immunocompetence, leading to decreased resistance to infection (Fish, 2008). Furthermore, studies in animal models have demonstrated a lower incidence and severity of sepsis in females compared to males, which may be attributed to the protective effect of estrogen (Sener et al., 2005; Marriott et al., 2006). However, further study is required to identify the specific mechanism of gender on the risk of in-hospital mortality after infection in patients with AIS and DM.

Various heart-related complications are also important causes of in-hospital mortality in AIS patients with DM after infection. In our study, previous MI was associated with a 1-fold increase, atrial fibrillation/flutter with a 70% increase, and chronic heart failure with a 60% increase of in-hospital mortality in DM patients who suffered from AIS and infection. The results indicated the lethality of the infection for patients with AIS and DM with cardiac diseases. Similar to the results of the current research, previous studies demonstrated that the severity of AIS (i.e., NIHSS score at admission) was independently associated with poor functional outcomes and in-hospital mortality after infection in patients with AIS (Aslanyan et al., 2004; Rocco et al., 2013; Suda et al., 2018).

In our study, elevated FPG at admission was an independent risk factor for in-hospital mortality after infection in AIS patients with DM. Furthermore, hyperglycemia on admission has been reported to be independently associated with poor outcomes and increased risk of death in AIS patients with or without DM (Williams et al., 2002; Kostulas et al., 2009; Poppe et al., 2009). Susceptibility to infection may partially explain the association between admission hyperglycemia and poor outcome of AIS. A large multicenter prospective randomized controlled trial showed that hyperglycemia is predictive of post-stroke infections in patients with AIS (Zonneveld et al., 2017). Moreover, admission hyperglycemia is associated with mortality in a multitude of infectious diseases, such as Coronavirus Disease 2019 (COVID-19), pneumonia, and central venous catheter-related bloodstream infection (Schuetz et al., 2014; Xie et al., 2020; Mamtani et al., 2021). Hyperglycemia aggravates inflammation and the oxidative stress response by damaging the brain-blood barrier, exacerbating brain edema, increasing apoptosis, disordering the coagulation function, and increasing plasma concentrations of free fatty acids, which may affect stroke outcomes after infection (Song et al., 2003; Yamato et al., 2007; Kruyt et al., 2010; Chiu et al., 2013; Fiorentino et al., 2013; Chen et al., 2022).

Remarkably, our study found that reperfusion therapy can increase the risk of in-hospital mortality in stroke patients with infection, while antiplatelet drug therapy reduces the risk of death. All the subjects included in our study had a history of diabetes, indicating the possibility that DM mediates the association between reperfusion therapy and the heightened risk of in-hospital mortality after infection. On the one hand, DM leads to suboptimal recanalization or hemorrhagic transformation after intravenous thrombolysis (Jiang et al., 2021). On the other hand, the inflammation in patients with infection is further amplified *via* the expression of cytokines triggered by up-regulated matrix metalloproteinase-9 (MMP-9), an inflammatory mediator induced by the synergy between DM and tissue-type plasminogen activator (tPA) (Jiang et al., 2021). Aspirin and clopidogrel, widely used as antiplatelet drugs in the fields of cardiovascular disease and stroke, have been reported to decrease the severity of infection and mortality in patients with sepsis (Gross et al., 2013; Valerio-Rojas et al., 2013). Aspirin plays an anti-inflammatory role by preventing the formation of pro-inflammatory cytokines and chemokines and reducing the migration and infiltration of neutrophils (Toner et al., 2015). Clopidogrel mitigates inflammation by antagonizing the P2Y12 receptor, a key factor in systemic inflammation in sepsis (Liverani et al., 2016). Patients who are not treated with antiplatelets generally have contraindications such as serious hemorrhage, and the weak constitution of such patients may be related to higher in-hospital mortality after infection.

Considering the lethality of stroke-associated infection for AIS patients with DM, especially elderly patients or those with many complications, prevention of infection is crucial. The results of two multicenter, randomized, open-label, blinded endpoint design, controlled trials published in The Lancet in 2015 showed that prophylactic antibiotics after AIS did not reduce the incidence of pneumonia and did not improve 3-month functional outcomes (Kalra et al., 2015; Westendorp et al., 2015). In Ulm et al. (2017) conducted a multicenter, randomized, controlled trial in which AIS patients with procalcitonin ultrasensitive (PCTus) >0.05 ng/ml (considered a possible bacterial infection) were treated with prophylactic antibiotics. The results showed no improvement in mRS function and no reduction in mortality at 90 days in the intervention group (Ulm et al., 2017). However, these randomized controlled trials were conducted in patients with AIS, while the patients with AIS and DM exhibited a higher rate of in-hospital mortality after infection. Therefore, these patients may benefit more from prophylactic antibiotic treatment, and further study is necessary to confirm this theory. However, appropriate measures should be taken for each patient to prevent infection regardless of the use of prophylactic antibiotics.

Nevertheless, this study has several limitations. First, stroke-associated infection was based on discharge diagnosis and did not exclude pre-stroke infection, which may bias the results. Second, antibiotic therapy could not be adjusted for due to data unavailability, which might influence the results of early outcomes and in-hospital mortality. Third, as our study population included only Chinese patients, the results might not be applicable to other populations. Finally, we only focused on the short-term effects of infection on AIS patients with DM, while the long-term effects need to be clarified *via* large-scale prospective studies in the future.

In summary, findings from this study reveal that infection is an independent risk factor of in-hospital mortality for patients with AIS and DM. Those patients require strengthened nursing management to prevent infection.

## Data availability statement

All analysis data are available with permission from the China National Clinical Research Center for Neurological Diseases. Further inquiries can be directed to the corresponding author.

## Ethics statement

The studies involving human participants were reviewed and approved by the Institutional Ethics Committee at Beijing Tiantan Hospital. Written informed consent for participation was not required for this study in accordance with the national legislation and the Institutional requirements.

## Author contributions

JX, MW, and YD: experimental design. MW, YJ, HG, RT, and DL: data curation. MW: writing—original draft. QinH, FY, XF, and YfL: data interpretation and validation. MW and TZ: visualization. JX and YD: writing—review and editing. JX, ZL, QingH, WG, and YhL: resources. All authors contributed to the article and approved the submitted version.

## Abbreviations

AIS: acute ischemic stroke
DM: diabetes mellitus
CSCA: Chinese Stroke Center Alliance
UTI: urinary tract infection
BMI: body mass index
MRI: magnetic resonance imaging
NIHSS: National Institutes of Health Stroke Scale
mRS: modified Rankin Scale score
SD: standard deviation
IQR: interquartile ranges
OR: odds ratio
CI: confidence interval
SBP: systolic blood pressure
TIA: transient ischemic attacks
IS: ischemic stroke
ICH: intracerebral hemorrhage
SAH: subarachnoid hemorrhage
MI: myocardial infarction
CRP: C-reactive protein
FPG: fasting plasma glucose
HCY: homocysteine
COVID-19: coronavirus disease 2019
PCTus: procalcitonin ultrasensitive.
